# The kinematic analysis of the lower limb during topspin forehand loop between different level table tennis athletes

**DOI:** 10.7717/peerj.10841

**Published:** 2021-03-12

**Authors:** Yuqi He, Xiang Lyu, Dong Sun, Julien S. Baker, Yaodong Gu

**Affiliations:** 1 Faculty of Sports Science, Ningbo University, Ningbo, China; 2 Department of Sport, Physical Education and Health, Hong Kong Baptist University, Hong Kong, Hong Kong

**Keywords:** Table tennis, Topspin forehand loop, Shot techniques, Kinematic

## Abstract

**Background:**

Topspin is one of the most attacking stroke in table tennis sport. The aim of this research was to investigate the kinematic characteristics of the lower limb (driving leg) during topspin forehand loop in different playing level table tennis athletes.

**Methods:**

Ten male table tennis athletes performed topspin forehand loop shots with maximal force to hit the ball that was played by a professional table tennis coach. The three-dimensional Vicon motion analysis system was used to capture the kinematic information.

**Results:**

The key findings from this research indicate that there were no significant differences in motion time between elite athletes (EA) and medium athletes (MA) during the entire phase (*P* = 0.784). EA showed significantly less knee (*P* < 0.001) as well as hip (*P* < 0.001) flexion in the BS stage when contrasted to MA, with a significant larger ankle varus (*P* = 0.003) as well as eversion (*P* < 0.001) than MA in the BS and FS phase, respectively. EA displayed a significant larger angular changing rate of ankle dorsiflexion (*P* < 0.001) and varus (*P* < 0.001) in the BS stage with ankle plantar flexion as well as eversion during the FS stage, with a significant larger ankle internal rotation (*P* = 0.003) and external rotation (*P* < 0.001) than MA in the BS and FS phase, respectively. Furthermore, EA showed significantly larger ankle dorsiflexion (*P* = 0.001) as well as plantarflexion (*P* < 0.001) ROM in the BS and FS phase respectively compared with MA.

**Conclusion:**

Ankle activities in the all plane displayed significant differences in kinematic characteristics between EA and MA. MA should pay attention to the function that ankle played in the kinetic chain, such as training the lower limb muscle rapid reaction ability to improve the energy transfer efficiency and capability of the kinetic chain.

## Introduction

Table tennis sport as one of the most popular and important racket sport in the world, there are at least 40 million competitive table tennis players in 2016 (Fuchs et al., 2018). The characteristics of the fast rotation and high speed in the topspin forehand loop make it become the most common and frequently used attacking technique in table tennis (Poizat, Thouvarecq & Seve, 2004). The topspin forehand loop was used frequently in table tennis winners compared with other types of stroke, and it is probably shows that mastery of this shot would make a critical effect to win matches (Malagoli Lanzoni, Di Michele & Merni, 2014). With the competition of table tennis becoming more and more intense, performing a better topspin forehand loop may influence the result of competitions. Mastering topspin forehand strokes could distinguish the performance of different level players even though it is difficult (Iino & Kojima, 2009).

Lower limbs played an important role to the performance during topspin forehand loop in racket sport, and the lower limb drive considerably influences ball and racket speed as the origin of the kinetic chain (Qian et al., 2016; Elliott, 2006; Seeley et al., 2011). A number of studies have investigated lower limb biomechanical contributions when playing table tennis and have analyzed details when using the topspin forehand loop. Malagoli Lanzoni et al. (2018) compared the kinematics of seven table tennis players using the long-line and cross-court topspin forehand loop. They mentioned a significantly larger flexion of the right knee at the moment of maximum racket velocity during the long-line. Iino & Kojima (2009) investigated the relation between different playing levels and kinematics information of racket during table tennis topspin stroke. They reported that high level players showed a significantly larger lower trunk rotation contribution to the racket speed at the moment of impact as well as a significantly larger maximum slope value in the speed-time curve of racket. They also tended to that the time for racket acceleration of advanced player was less than the intermediate players. Qian et al. (2016) compared biomechanics information of lower limbs between different level table tennis player during the forehand loop. They reported that compared with intermediate players. The superior players showed a significantly larger external rotation of the knee joint as well as flexion of the hip joint in the backward-end, and a significantly larger hip internal rotation and extension was showed at the forward-end. The above research shows that, investigations into the lower limbs biomechanical characteristics during the topspin stroke are crucial and necessary.

As one of the most offensive table tennis stroke, topspin forehand is extremely important for an aggressive player to master this shot properly (Iino & Kojima, 2009). Iino & Kojima (2009) reported the biomechanical analysis during topspin forehand loop against backspin balls between different level table tennis players. They reported values for the kinematic analysis of lower trunk flexion, rotation, and extension. Two group male table tennis players that include nine advanced and eight intermediate hit topspin forehands to against heavy and light backspins. However, the ankle, hip and knee kinematics in the frontal, sagittal and transverse planes were not investigated in their research. Hence, he aim of this research was to show the knee, hip and ankle joint kinematic characteristics in sagittal, frontal and transverse plane during a set of topspin forehand loop hits in different playing level table tennis players. The motion time, ROM, angular changing rate and joint angle of the lower limb in sagittal, frontal and transverse plane were measured and analysis in this research. From a practical point of view, this research would provide useful information in the modulation of body motion requirements to table tennis athlete. Previous studies have described the torsional mechanism of topspin hitting in table tennis players at different levels. However, as the part of lower limb kinetic chain, the mechanism and differences of lower limb joints have not been studied in detail, the hypothesis of this research was that the lower limb especially driving leg as the basic section of power chain will prevent a significant difference between two different level table tennis athletes.

## Methods

### Participants

As outlined in Table 1. Ten professional male table tennis athletes were allowed to participate in this study. All of the participate was attached to the table tennis team at Ningbo University, Ningbo China. The group of participant was divided into two groups: Five players (Height: 173 ± 4.2 cm, Weight: 70 ± 7.9 kg, Experience: 10 ± 3 years) were belong to elite athletes (EA) who play in the China National Level I. Another five players (Height: 172 ± 2.7 cm, Weight: 69 ± 8.5 kg, Experience: 9 ± 3 years) were belong to medium athletes (MA) who play in China National Level II. All participants were right-handed and nobody with previous diseases or deformity of the lower limb for 3 months before this study. The handedness of the athletes was identified and confirmed based on the preferential hand used to hold the racket. Caffeine was forbidden to ingestion of all participant for 4 h before this study. Before the commencement of this research, participants were provided to write informed consent. This research was approved by the Ethics Committee of Research Academy of Grand Health at Ningbo University (RAGH20191121).

**Table 1:** The information of the participant (Mean ± SD).

Population	Level	Height (cm)	Weight (kg)	Handedness	Experience (year)
5	EA	173 ± 4.2	70 ± 7.9	Right	10 ± 3
5	MA	172 ± 2.7	69 ± 8.5	Right	9 ± 3

### Experimental procedures

**Figure 1:**
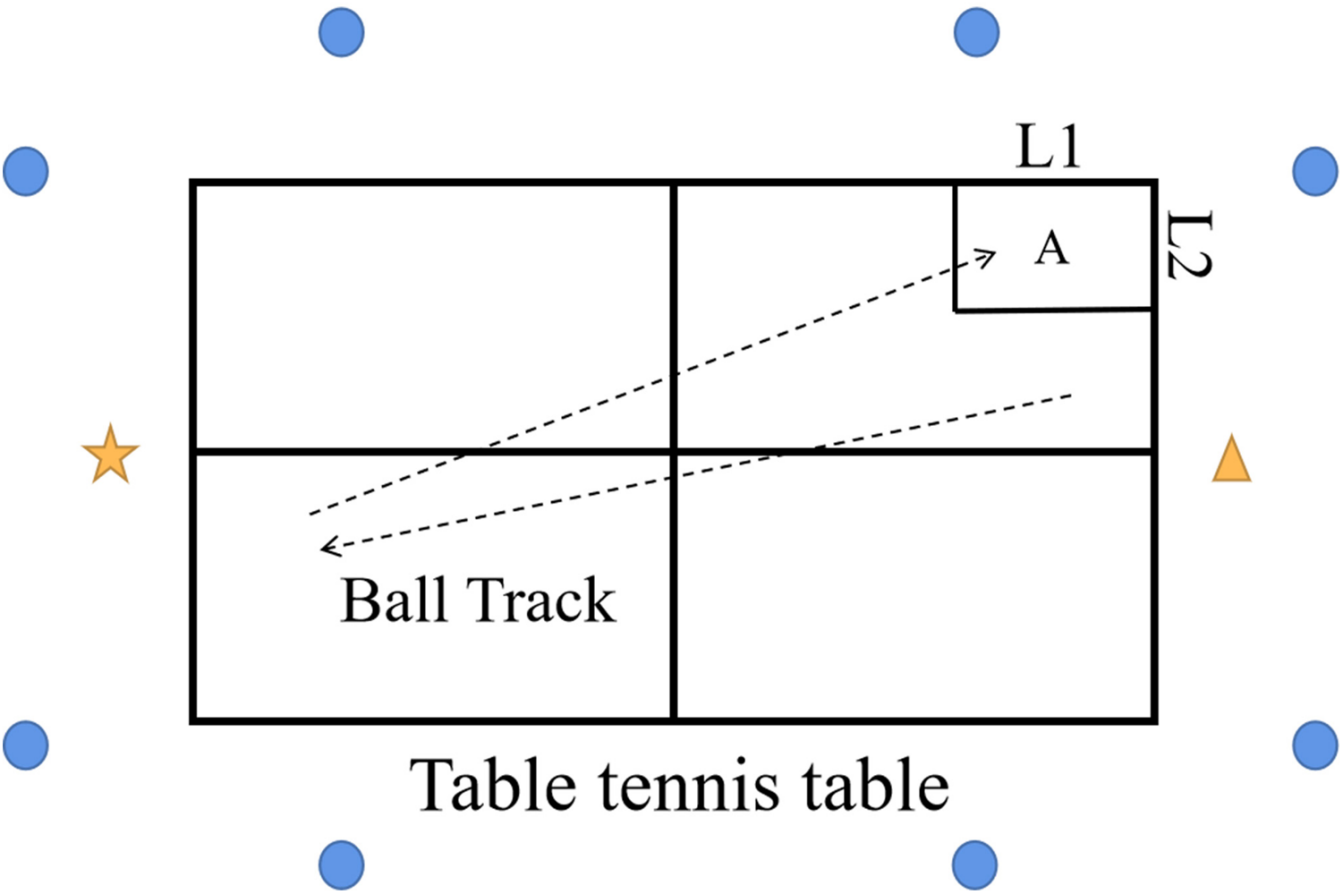
Experiment setup. L1: 45 cm, L2: 38 cm, A: Target area, ☆: Participant, Δ: Coach, ○: Camera.

As outlined in Fig. 1. Data were collected as previously described in Qian et al. (2016) and Wang et al. (2018). The kinematic information with participant was captured by an 8-camera Vicon motion analysis system (Oxford Metrics Ltd., Oxford, UK) at a 100 Hz frequency. A total of 16 reflective markers (diameter: 14 mm) were attached with adhesive tape on the bilateral lower limbs respectively. Marker locations included: posterior-superior iliac spine, anterior superior iliac spine, lateral mid-thigh, lateral knee, lateral malleolus, lateral mid-shank, second metatarsal head, and calcaneus.

Players were asked to perform only the forehand topspin loop with maximal power to return the topspin ball played from a professional table tennis coach to the target area (A: L1 = 45 cm, L2 = 38 cm ). If the ball was missed or outside of the target area (A), the action will not be measured, and participants need to do it again. The coach was asked to keep a stable ball track and drop point, the speed and frequency of the ball was also controlled by the coach. The action of the players was recorded until three full motions were successfully captured. And participant was allowed adjustment or rests 1 min between actions. In addition, all players were given at least 15 min to warm up and adaption the experimental environment before the commencement of the official experiment and data collection.

### Segmental coordinate systems

The segmental coordinate systems were created as previously described in Iino & Kojima (2009).

#### Lower trunk

The vector that from the middle point on both sides of the hip joint centers to the middle point on both side of shoulder joint centers was defined as the *z*-axis of the lower trunk coordinate system. The *X*-axis was defined as the cross product of the vector from the center of the right hip to the center of the left hip with the *z*-axis. The cross product of the *z*-axis and *x*-axis was defined as *y*-axis.

#### Driving leg

The *z*-axis of the driving leg (right leg) was same as the lower trunk *z*-axis. The *y*-axis was the cross product of the *z*-axis of the system and a vector from the ankle joint center to the knee joint center. The cross product of the *y*- and *z*-axis was defined as *x*-axis.

#### Ankle

The *y*-axis of the ankle coordinate system was a vector from the head of the third phalangeal to the center of the ankle joint. The *x*-axis was a vector from the first phalanx medial to the fifth phalanx lateral. The *z*-axis was the cross product of the *z*- and *x*-axes of the ankle.

### Experimental material

The location of the experiment was at Ningbo University table tennis training Centre, which is a professional competition and training facility. During the experiment, players were asked to wear professional table tennis matches shoes. Besides, whole of the player was asked to use the same table tennis racket (Timoboll-zlc; Butterfly Technical Center, Tokyo, Japan) with the Butterfly Tenergy 05 Max (Butterfly Technical Center, Tokyo, Japan) and DHC Hurricane 3 (Double Happiness Sports Company, Shanghai, China) rubber sheets. The DHC Hurricane 3 rubber was the forehand side one. And the playing table used for data capture (Rainbow; Double Happiness Sports Company, Shanghai, China) was a professional game table.

### Definition motion phase

**Figure 2:**
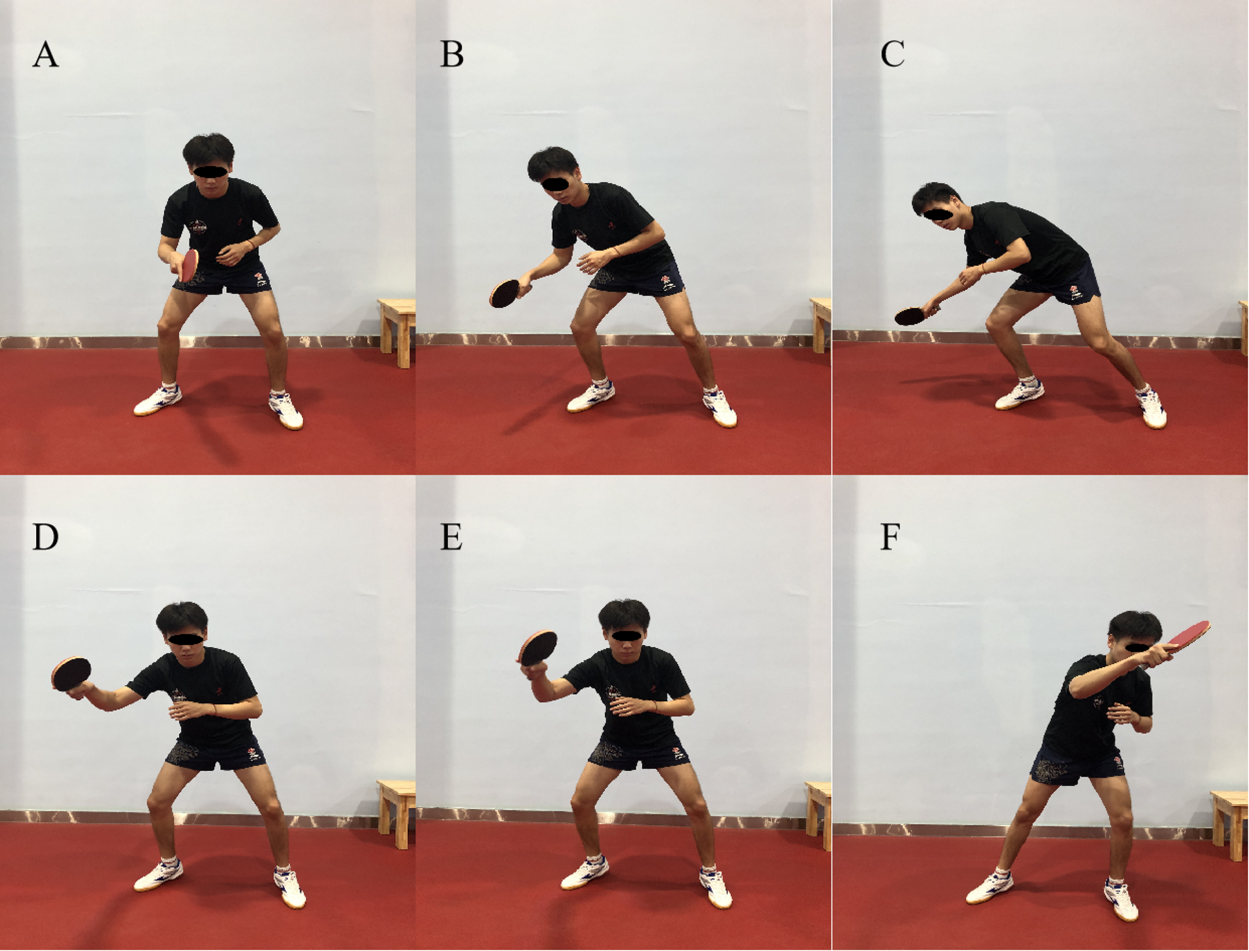
The divide and definition of motion phase. Motion phase A was defined as a natural position (NP), (A–C) was defined as the backswing phase (BS), (D–F) was defined as the forward swing phase (FS). Position C was defined as the key event which meant the end of the backswing phase (EB). Position F was defined as the key event which meant the end of the forward swing phase (EF).

As outlined in Fig. 2. Motion phase A was defined as a natural position (NP). Figures 2A–2C was defined as the backswing phase (BS), Figs. 2D–2F was defined as the forward swing phase (FS). Besides, this research focus on the key event of the entire motion, so we defined the position C as the key event which meant the end of the backswing phase (EB). And the position F was defined as the key event which means the end moment of the stage of the forward swing (EF).

### Statistical analysis

Statistical analysis and calculation were used by SPSS 19.0 version software (SPSS Inc., Chicago, IL, USA). The normal distribution of variables was verified using the Shapiro-Wilks normality test. As the driven leg, the right leg kinematic differences in the topspin forehand loop between the two levels of players were examined by independent *T*-tests. The Analysis included joint angles, motion time, angular changing rate and ROM of the ankle, knee, and hip joint. The significance level was set at *P* < 0.05.

## Results

### Motion time

As outlined in Table 2. The time taken to perform a topspin loop was 0.96 ± 0.09 s and 0.97 ± 0.09 s for EA and MA, respectively. EA demonstrated significantly less time than MA in the BS stage (*t*-value = −3.097, *P* = 0.004), however, EA showed a significantly larger time in the FS phase (*t*-value = 2.180, *P* = 0.038). Moreover, there were no significant differences in the time during the entire stage between EA and MA (*t*-value = −0.277, *P* = 0.784).

**Table 2:** Comparison of time at the phase of BS and FS between EA and MA (unit: second).

Variables	EA	MA	*P*-Value
	Mean ± SD	Mean ± SD	
BS phase	0.39 ± 0.06	0.45 ± 0.05	0.004*
FS phase	0.57 ± 0.07	0.52 ± 0.06	0.038*
Entire phase	0.96 ± 0.09	0.97 ± 0.09	0.784

**Notes:**

* A significant difference at the EA and MA.

BS, backward-swing; FS, forward-swing; EA, elite athlete; MA, medium athlete.

### Lower-limb joint angle

**Figure 3:**
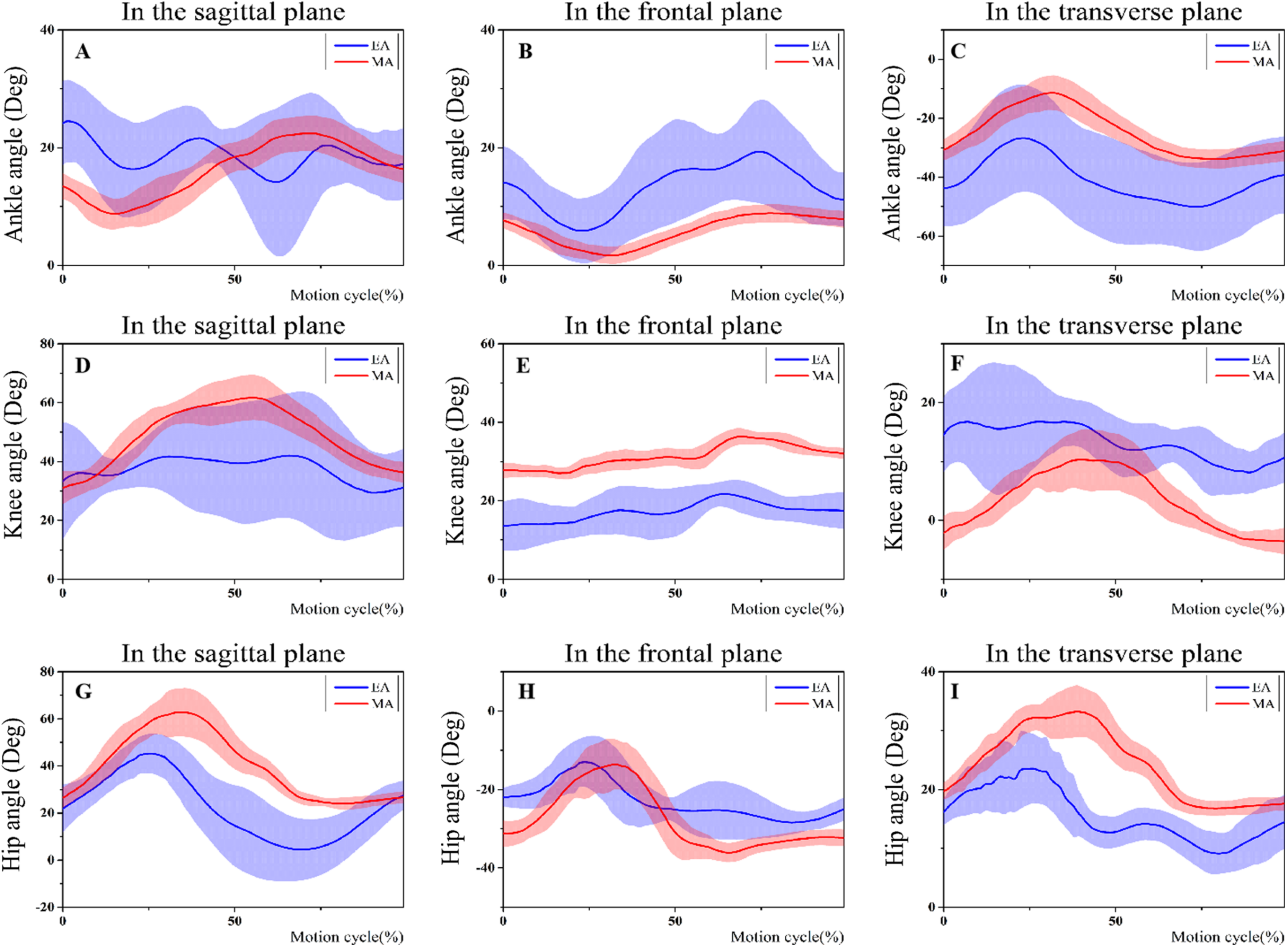
Changes of the lower limb joints angle during the entire phase in three planes. (A–C) Ankle angle changes during the entire phase in three planes; (D–F) knee angle changes during the entire phase in three planes; (G–I) Hip angle changes during the entire phase in three planes.

Table 3 and Fig. 3 showed the angles of joint at BE as well as FE in the transverse, frontal, as well as sagittal planes for both EA with MA. In the frontal plane as well as the transverse plane, EA displays significant differences of joint angles for the entire stage compared with the MA. In the sagittal plane, EA showed significantly less knee (*t*-value = −7.496, *P* < 0.001) and hip (*t*-value = −25.397, *P* < 0.001) flexion in the BE phase compared with MA. In the frontal plane, EA showed a significantly larger ankle varus (*t*-value = 3.282, *P* = 0.003) and eversion (*t*-value = 8.799, *P* < 0.001) than MA in the BE and FE phases, respectively. Moreover, in the transverse plane, EA displayed a significantly larger ankle internal rotation (*t*-value = −3.320, *P* = 0.003) and external rotation (*t*-value = −7.428, *P* < 0.001) than MA in the BE and FE phase, respectively. EA showed a significantly larger knee external rotation (*t*-value = 5.027, *P* < 0.001) and internal rotation (*t*-value = 19.219, *P* < 0.001) in the BE and FE phase respectively compared with MA. However, MA showed a significantly larger hip external rotation (*t*-value = −6.299, *P* < 0.001) and internal rotation (*t*-value = −10.590, *P* < 0.001) in the BE and FE phase respectively compared with EA.

**Table 3:** Comparison of joints angles at key events between EA and MA (unit: degrees).

Variables	ANKLE		KNEE		HIP	
	BE	FE	BE	FE	BE	FE
	Mean ± SD	Mean ± SD	Mean ± SD	Mean ± SD	Mean ± SD	Mean ± SD
X (EA)	15.77 ± 5.12*	15.96 ± 13.52	41.15 ± 8.83*	42.50 ± 22.52	48.31 ± 2.13*	2.00 ± 11.29*
X (MA)	12.34 ± 3.27*	22.13 ± 2.37	58.80 ± 2.29*	47.86 ± 5.00	66.48 ± 1.77*	23.35 ± 2.28*
Y (EA)	4.85 ± 3.78*	17.42 ± 3.59*	14.74 ± 2.86*	20.73 ± 3.28*	−10.64 ± 3.12	−26.05 ± 7.36*
Y (MA)	1.55 ± 0.97*	8.74 ± 1.31*	30.18 ± 2.12*	36.01 ± 2.14*	−11.88 ± 2.18	−34.07 ± 2.45*
Z (EA)	−25.25 ± 16.53*	−50.60 ± 8.36*	18.86 ± 5.94*	13.61 ± 1.96*	23.60 ± 6.41*	11.37 ± 1.41*
Z (MA)	−10.74 ± 3.70*	−33.67 ± 2.82*	10.96 ± 1.33*	−0.10 ± 2.20*	34.18 ± 1.13*	16.40 ± 1.18*

**Notes:**

* A significant difference at the hip, knee, and ankle (respectively) (*P* < 0.05)

X, the sagittal plane; Y, the frontal plane; Z, the transverse plane. BE, backward-end; FE, forward-end; EA, elite athlete; MA, medium athlete.

### Range of motion

**Figure 4:**
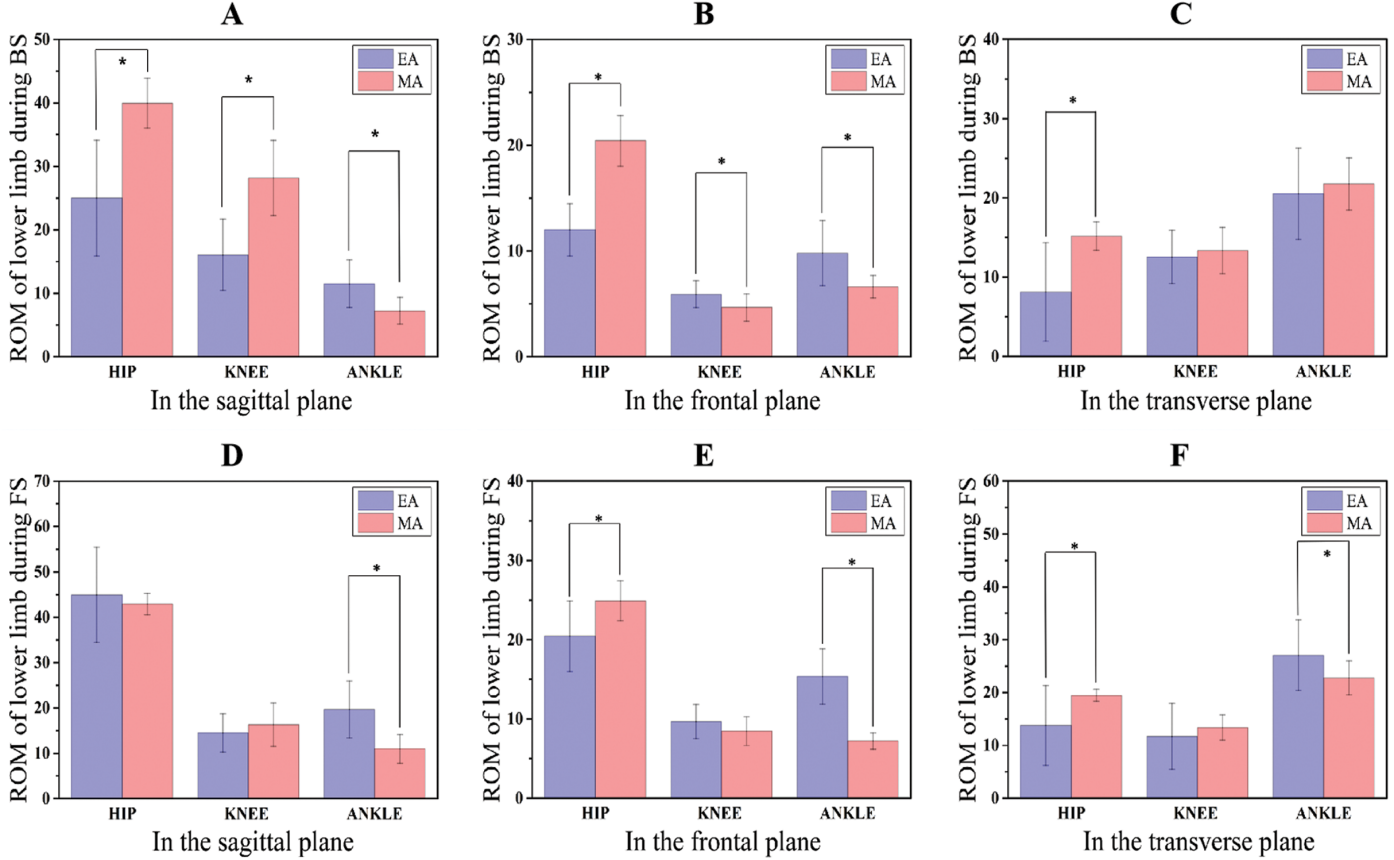
Changes of lower limb ROM during BS and FS phase in three planes. (A–C) Lower limb ROM changes during BS in three planes; (D–F) lower limb ROM changes during FS in three planes.

ROM at BS as well as FS stage between EA and MA in all planes have displayed at the Table 4 as well as Fig. 4. Lower-limb ROM showed significant differences during the BS as well as FS phases between EA and MA. Compared with MA, EA showed significantly larger ankle dorsiflexion (*t*-value = 3.838, *P* = 0.001) and plantarflexion (*t*-value = 4.792, *P* < 0.001) ROM in the BE and FE phase respectively. Moreover, EA showed a significantly larger ankle varus (*t*-value = 3.788, *P* = 0.001) and external rotation (*t*-value = 2.251, *P* = 0.032) ROM in the BE and FE phase respectively. However, EA showed significantly less hip flexion (*t*-value = −5.836, *P* < 0.001) and external rotation (*t*-value = −4.211, *P* < 0.001) ROM in the BE phase contrast with MA.

**Table 4:** Comparison of ROM at the phase of BS and FS between EA and MA (unit: degrees).

Variables	ANKLE		KNEE		HIP	
	BS	FS	BS	FS	BS	FS
	Mean ± SD	Mean ± SD	Mean ± SD	Mean ± SD	Mean ± SD	Mean ± SD
X (EA)	11.50 ± 3.76*	19.66 ± 6.31*	16.02 ± 5.62*	14.48 ± 4.25	25.01 ± 9.10*	44.93 ± 10.52
X (MA)	7.23 ± 2.11*	10.95 ± 3.13*	28.16 ± 5.92*	16.31 ± 4.79	39.94 ± 3.92*	42.90 ± 2.32
Y (EA)	9.78 ± 3.08*	15.36 ± 3.47*	5.90 ± 1.28*	8.44 ± 1.80	12.00 ± 2.47*	20.44 ± 4.45*
Y (MA)	6.60 ± 1.06*	7.22 ± 1.03*	4.65 ± 1.28*	15.36 ± 3.47	20.43 ± 2.40*	24.90 ± 2.51*
Z (EA)	20.52 ± 5.77	27.05 ± 6.66*	12.54 ± 3.37	11.67 ± 6.25	8.11 ± 6.23*	13.74 ± 7.51*
Z (MA)	21.73 ± 3.31	22.75 ± 3.20*	13.34 ± 2.91	13.37 ± 2.40	15.16 ± 1.80*	19.45 ± 1.14*

**Notes:**

X, the sagittal plane; Y, the frontal plane; Z, the transverse plane. BS, backward-swing phase; FS, forward-swing phase; EA, elite athlete; MA, medium athlete.

* A significant difference at the hip, knee, and ankle (respectively) (*P* < 0.05).

### Angular changing rate

**Figure 5:**
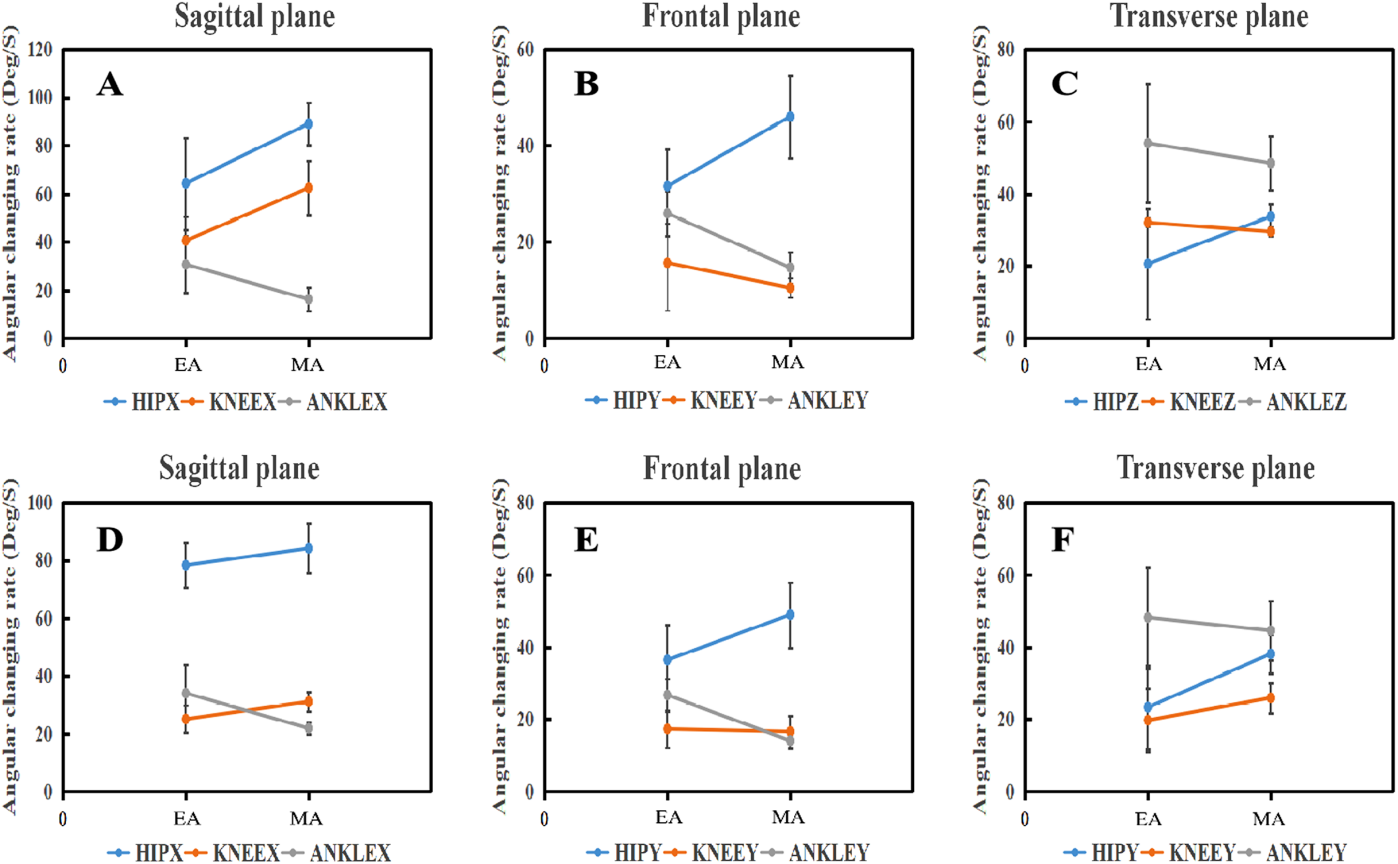
Angular changing rate of lower limb joints during BS and FS phase in three planes. The top is the BS phase, Bottom is the FS phase. (A–C) Angular changing rate of lower limb during BS phase in three planes; (D–F) angular changing rate of lower limb during FS phase in three planes.

Angular changing rates at BS as well as FS phases between EA and MA in all planes were shown at Table 5 as well as Fig. 5. In the stage of BS and FS, both in the sagittal and frontal plane, the angular changing rate of ankle joint for the EA was significantly larger than MA. However, EA showed a significantly smaller angular changing rate in the hip (*t*-value = −4.572, *P* < 0.001) and knee (*t*-value = −5.592, *P* < 0.001) joint during BS in the sagittal plane.

**Table 5:** Comparison of the angular changing rate at the phase of BS and FS between EA and MA (unit: degrees/second).

Variables	ANKLE		KNEE		HIP	
	BS	FS	BS	FS	BS	FS
	Mean ± SD	Mean ± SD	Mean ± SD	Mean ± SD	Mean ± SD	Mean ± SD
X (EA)	34.01 ± 11.80*	30.98 ± 8.48*	37.56 ± 10.15*	25.06 ± 5.68*	55.02 ± 19.10*	72.26 ± 14.85
X (MA)	15.09 ± 4.98*	21.81 ± 8.05*	59.60 ± 11.30*	31.44 ± 6.71*	89.62 ± 8.79*	77.66 ± 12.90
Y (EA)	23.74 ± 9.93*	26.22 ± 4.61*	15.89 ± 4.64*	16.22 ± 5.23	33.84 ± 7.70*	34.07 ± 9.74*
Y (MA)	14.86 ± 2.08*	13.70 ± 2.21*	10.99 ± 3.33*	16.50 ± 4.47	46.23 ± 8.57*	46.70 ± 9.04*
Z (EA)	55.99 ± 16.37	47.63 ± 13.89	31.03 ± 5.63	18.66 ± 8.91*	10.46 ± 15.21*	15.97 ± 11.52*
Z (MA)	46.39 ± 7.50	43.07 ± 8.23	28.81 ± 4.93	25.73 ± 4.15*	34.19 ± 3.32*	35.53 ± 5.44*

**Notes:**

* A significant difference at the hip, knee, and ankle (respectively) (*P* < 0.05).

X, the sagittal plane; Y, the frontal plane; Z, the transverse plane. BE, backward-swing phase; FE, forward-swing phase; EA, elite athlete; MA, medium athlete.

## Discussion

The aim of the research was to describe and compare the lower limb kinematic characteristics of the topspin forehand loop between EA and MA. The key findings of this study were: In time-spending, have no significant differences between EA as well as MA during the entire playing phases. (1) EA showed a significantly less knee and hip flexion in the BS stage compared with MA, and a significantly larger ankle varus and eversion than MA in the BS and FS phases, respectively. (2) EA displayed a significantly larger angular changing rate in ankle dorsiflexion as well as varus during the BS phase with ankle plantarflexion and eversion during the FS phase. Moreover, EA showed a significantly larger ankle internal and external rotation than MA in the BS and FS phases, respectively. (3) Compared with MA, EA showed a significantly larger rotation of external as well as internal in the knee in the BS and FS stage respectively. EA showed significantly larger ankle dorsiflexion and plantarflexion ROM in the BS and FS phases respectively compared with MA.

Between EA and MA during the entire phase, have no differences in motion time for significant. However, significantly less time was showed by EA during the BS phase as well as a significantly larger time period during FS compared with MA. Bankosz & Winiarski (2017) reported that increasing the BS phase resulted in longer subsequent phases and elongation of the total time. However, Qian et al. (2016) reported that compared with intermediate players, the superior players showed less time during FS.

Elite athletes showed a significantly larger ankle varus and internal rotation than MA in the BS stage. This could reinforce the stretching activity of internal rotation, and resulting in the contraction effects enhanced during the FS phase (Zhang et al., 2017). According to the theory of stretch-shortening cycle, performance of concentric contraction of muscle tendons would be enhanced by the elastic energy stored in the process of eccentric (Elliott, 2006; Walshe, Wilson & Ettema, 1998). This means that EA makes a greater preparation of the ankle during BS compared with MA. Compared with MA, EA showed significantly less knee and hip flexion in the BS phase, as well as a significantly larger ankle varus and internal rotation in the BS phase. This probably indicates the compensatory mechanism of the ankle joint in the BS phase. Excessive flexion of the hip and knee joint may result in the next lower limb stretching action initial speed to decrease. More sufficient torsion of the ankle joint can compensate for the flexion of the knee and hip joint, which is helpful to improve the flexibility of the lower limbs. In fact, a way that a high racquet speed could generate on impact without undue injury risk was must coordinate in the all kinetic chain links (Elliott, Grove & Gibson, 1988; Elliott, 2006).

In the stage of FS, EA showed a significantly larger ankle external rotation and eversion than MA. Moreover, EA displayed a significantly larger external rotation and internal rotation of the knee in the BS and FS phases respectively compared with MA. This may result in a greater transfer range of weight to promote momentum generation (Ball & Best, 2007). Advanced players exhibited more whole-body movements than lower-skilled players by rotating the upper body through effective use of the knee joints that in the previous table tennis studies on lower-limb biomechanics (Bankosz & Winiarski, 2018a). Myers et al. (2008) reported that increased rotational counter movement of torso and pelvic at the top of downswing in golf was associated with increased ball velocity. Compared with MA, EA showed a more sufficient ankle eversion rotation in the FS phase, and EA more sufficient ankle pedaling and stretching probably means a faster weight transfer effect. Bankosz & Winiarski (2018b) have reported that involved segments of the lower limbs support proximal-to-distal movement sequencing: plantarflexion and rotation of the ankle joint, and rotation in the knee and hip joints. These movements result in the upward and forward velocity of the whole playing upper limb increased at the contact moment during the follow-through movement. Moreover, Kasai & Mori (1998) have evaluated the technique and performance of topspin shots. They drew attention to the differences between players, depending on the performance level. Players with a high-performance level had significant cooperation from the whole body, specifically the rotation of the trunk and the work of the knee joints. According to kinetic chain perspectives, the speed of racket and ball in racket sport considerably influenced by the energy transferred from lower-limb to the upper limbs (Lam et al., 2018; Reid, Elliott & Alderson, 2008). An important factor related to optimizing energy transfer in the kinetic chain is joint angular velocity which is expected to increase as skill levels improve (Seeley et al., 2011). Another key finding from this study is that EA displayed a significantly larger ankle dorsiflexion and varus changing rate during the BS phase with ankle plantarflexion and eversion during the FS phase. Qian et al. (2016) have reported a similar result. This finding further reveals that the ankle joint as the starting point of movement plays an important role in topspin forehand loop. The increased ankle angular changing rate of EA during FS in this study may be related to a more effective lower limb drive the ball speed to increase (Qian et al., 2016). The quality of whiplash-like action was assessed by the sequence as well as interval of time in momentum transfer between the distal and proximal (Kibler & Van der Meer, 2001). The higher angular changing rate of the ankle probably means compared with MA, EA displayed a faster weight transfer and a shorter time of pedal and stretch in the lower limb. This proximal segments power transference may play a crucial role of throwing speed, such as the ability that mechanical energy transmits from the trunk to the upper limb to produce a faster racket speed in the athletes (Zemkova, Muyor & Jelen, 2018). Wang et al. (2018) have reported the similar point, in their study, EA presented a larger angular changing rate of lower limb joint, and less time to hit the table tennis ball, that probably means a higher speed of play. Seeley et al. (2011) revealed that the speed of post-impact increased from slow to medium levels results in the velocity of peak ankle plantarflexion and hip extension increased in racket sport forehand. However, during the forehand topspin, the larger knee and ankle loading magnitudes found in sidestep and cross-step footwork would predispose to overuse conditions of table tennis players such as jumper knee (Lam et al., 2018; Rajabi et al., 2012) and ankle sprain injuries (Lam et al., 2018; Fong et al., 2009). This requires to development the ability of rapid muscle response to stabilize dynamic joint during sport activities (Cerqueira et al., 2020). We can speculate that a focus on the rapid response ability of the muscles that surrounding the ankle joint will effectively enhance the ankle dynamic stability as well as decrease the injury likelihood in the ankle during the fast movement of table tennis topspin forehand loop play. This information will guide coaches and athletes to attach importance to the role of the ankle joint in the lower limb dynamic chain in the forehand topspin, and the training of the lower limb muscle rapid reaction ability, especially the ankle joint.

There are some limitations to this study that should be mentioned. One of the major limitations of this study was that the athletes performed the action without a match environment. Besides, during the moment of racket-ball impact, there are no variables information in this study.

## Conclusion

The forehand technique between EA and MA athletes in the lower limb kinematics was investigated with quantitative analysis in this research. It comprehensively shows the lower limb kinematic characteristics and differences between EA and MA athletes in table tennis topspin techniques. The activity of ankle in the plane of sagittal, frontal and transverse displayed the most significant differences in kinematic characteristics between EA and MA. Besides, MA should pay full focus on the function that ankle played in the kinetic chain. This will improve the energy transfer efficiency and capability of the kinetic chain. The findings of this study can provide information about improved topspin technical for coaches and athletes.

## Supplemental Information

10.7717/peerj.10841/supp-1 Supplemental Information 1 Raw data exported from the lower lime angle of elite athletes and medium athletes during entire phase for the detailed investigation shown Fig. 3.

10.7717/peerj.10841/supp-2 Supplemental Information 2 Raw data exported from the lower lime angle of elite athletes and medium athletes during entire phase for the detailed investigation shown Table 3.

10.7717/peerj.10841/supp-3 Supplemental Information 3 Raw data exported from the angular changing rate and ROM during BS and FS phase for the detailed investigation shown Figs. 4, 5, and Tables 4, 5.

10.7717/peerj.10841/supp-4 Supplemental Information 4 Raw data exported from the angle of key event during BS and FS phase for the detailed investigation shown Table 3.

10.7717/peerj.10841/supp-5 Supplemental Information 5 Raw data exported from the time of motion during BS, FS and entire phase for the detailed investigate shown Table 2.

10.7717/peerj.10841/supp-6 Supplemental Information 6 Participant demographics.

## Additional Information and Declarations

### Data Availability

The following information was supplied regarding data availability: Raw measurements are available in Supplemental Files.
